# A nearly fatal primary Epstein-Barr virus infection associated with low NK-cell counts in a patient receiving azathioprine: a case report and review of literature

**DOI:** 10.1186/s12879-019-4022-3

**Published:** 2019-05-10

**Authors:** Minna Honkila, Riitta Niinimäki, Mervi Taskinen, Outi Kuismin, Kaisa Kettunen, Janna Saarela, Sami Turunen, Marjo Renko, Terhi Tapiainen

**Affiliations:** 1 Department of Children and Adolescents, Oulu University Hospital, P.O. Box 23, FIN-90029 OYS, Oulu, Finland; 2 PEDEGO Research Unit and Medical Research Center Oulu, University of Oulu, Oulu, Finland; 3 Division of Pediatric Hematology, Oncology and Stem Cell Transplantation, Helsinki University Hospital, Helsinki, Finland; 4 Department of Clinical Genetics, Oulu University Hospital, Oulu, Finland; 5 Institute for Molecular Medicine Finland, University of Helsinki, Helsinki, Finland

**Keywords:** Epstein-Barr virus infections, Azathioprine, Killer cells, natural, Whole exome sequencing, Rituximab

## Abstract

**Background:**

Symptomatic primary Epstein-Barr virus infection is a usually self-limiting illness in adolescents. We present a case of an adolescent who had been receiving azathioprine for inflammatory bowel disease for four years and developed a life-threatening primary Epstein-Barr virus infection successfully treated with rituximab.

**Case presentation:**

An 11-year-old girl presented with chronic, bloody diarrhea. Endoscopic biopsies confirmed a diagnosis of chronic ulcerative colitis with features of Crohn’s disease. Azathioprine was initiated after one year due to active colitis. She responded well and remission was achieved. At the age of 16 years she developed a life-threatening Epstein-Barr virus infection including severe multiple organ failure and was critically ill for 4 weeks in the intensive care unit. Natural killer cells were virtually absent in the lymphocyte subset analysis. Azathioprine was stopped on admission. She was initially treated with corticosteroids, acyclovir and intravenous immunoglobulin. Approximately 30 days after admission, she developed signs of severe hepatitis and pneumonitis and received weekly rituximab infusions for 8 weeks. Primary immunodeficiency was excluded by whole exome sequencing in two independent laboratories. Persistent viremia stopped when the natural killer cell count started to rise, approximately 90 days after the cessation of azathioprine.

**Conclusions:**

We found 17 comparable cases in the literature. None of the previous cases reported in the literature, who had been treated with azathioprine and developed either a severe or a fatal Epstein-Barr virus infection, underwent full genetic and prospective immunological workup to rule out known primary immunodeficiencies. Recently, azathioprine has been shown to cause rather specific immunosuppression, resulting in natural killer cell depletion. Our case demonstrates that slow recovery from azathioprine-induced natural killer cell depletion, 3 months after the stopping of azathioprine, coincided with the clearance of viremia and clinical recovery. Finally, our choice of treating the patient with rituximab, as previously used for patients with a severe immunosuppression and Epstein-Barr virus viremia, appeared to be successful in this case. We suggest testing for Epstein-Barr virus serology before starting azathioprine and measuring natural killer cell counts during the treatment to identify patients at risk of developing an unusually severe primary Epstein-Barr virus infection.

## Background

Symptomatic primary Epstein-Barr virus (EBV) infection is a usually self-limiting illness presenting with fever, lymphadenitis, tonsillitis, hepatitis, and splenomegaly in adolescents and young adults. The duration of EBV viremia during primary EBV infection is rather short, while the median half-time for viral elimination is three days [1]. It is well known that after organ or allogenic hematopoietic stem cell transplantation (HSCT), or in patients with primary immunodeficiency, EBV primary infection or reactivation can cause uncontrolled and severe infection and lymphoproliferation. We present a case of an adolescent who had been receiving azathioprine for inflammatory bowel disease for four years who developed a life-threatening EBV infection. In addition, we performed a systematic review of the literature on azathioprine and EBV infection.

## Case presentation

### Methods

**Fig. 1:**
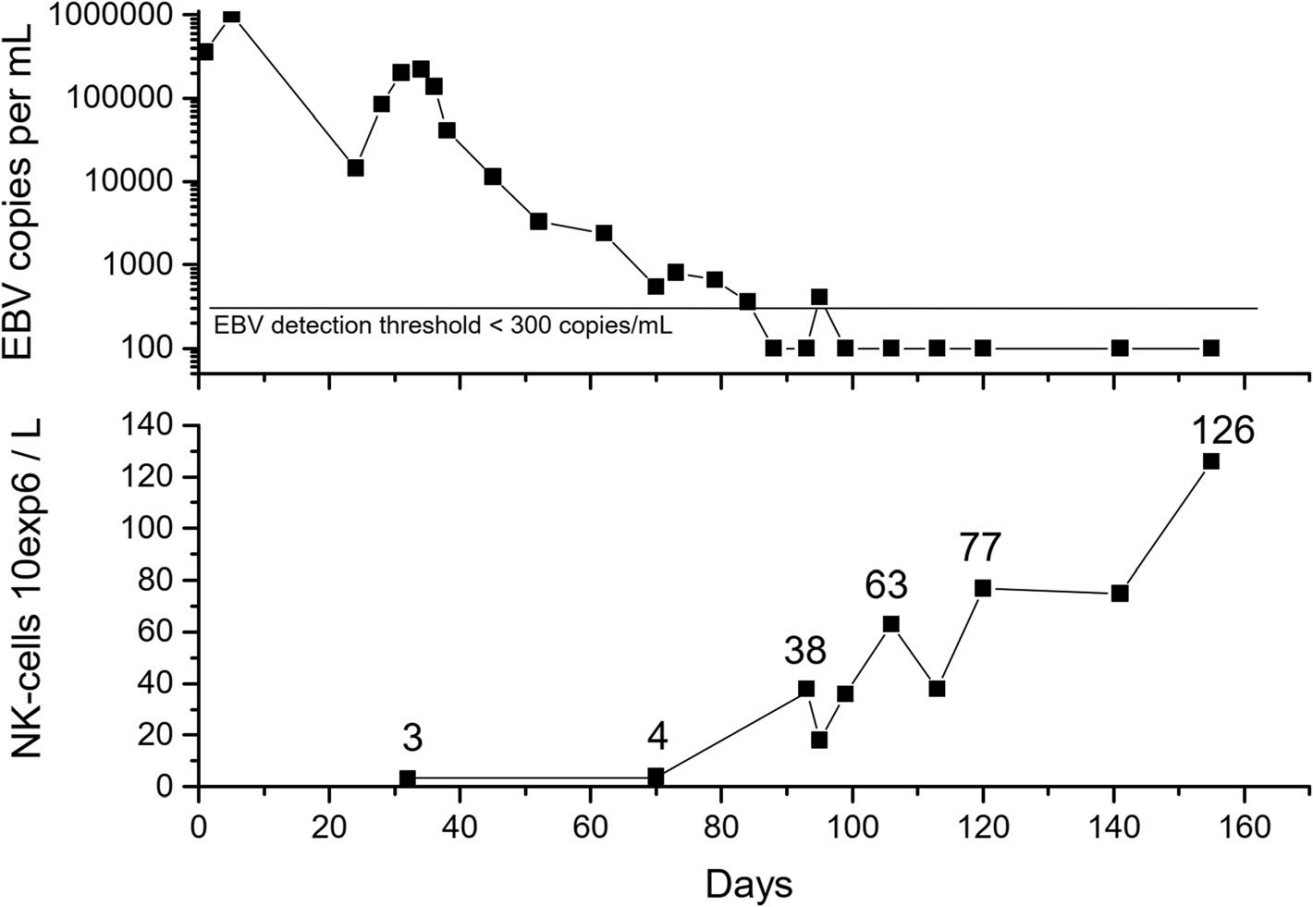
Epstein-Barr viremia and NK-cell counts after admission in a critically ill adolescent with a primary EBV infection and previously treated with azathioprine for inflammatory bowel disease. Azathioprine was stopped on day 1. NK-cell counts started to rise after 90 days and were normal after 120 days, coinciding with clearance of the viremia. During the course of illness, CD8+ T-cell counts were 1131 (220–1129 × 10^6^/L) on day 32, 2535 on day 70, 2487 on day 93 and 2537 on day 106

We first followed our patient up closely during severe EBV infection using repeated flow cytometry measurements to detect all lymphocyte subpopulations, including natural killer (NK) cell counts. The EBV viral load was measured using a quantitative nucleic acid amplification test (Fig. 1). Immunoglobulin (Ig) M antibodies against viral capsid antigen (VCA) and IgG antibodies to EBV nuclear antigen (EBNA) were measured. Whole exome sequencing (WES) was performed in two independent laboratories, the Institute for Molecular Medicine, Helsinki, Finland, as previously described [2], and Centogene, Germany (www.centogene.com), in order to detect any known primary immunodeficiency. Both the patient and her mother gave their written informed consent to the publication of this case history. We then performed a systematic literature search via PubMed in December 2016 using the terms “Epstein-Barr virus” and “azathioprine”. A total 154 possible papers were identified for review, of which 13 were retrieved for detailed examination.

### The patient

An 11-year-old girl presented with chronic, bloody diarrhea. Endoscopic biopsies confirmed a diagnosis of chronic ulcerative colitis with features of Crohn’s disease. She was treated first with prednisolone and mesalazine, but azathioprine, 75 mg/day (2.5 mg/kg), was started after one year on account of continued active colitis. She responded well to this and remission was achieved. At the age of 16 years she was admitted to hospital on account of a high fever and poor general condition. Azathioprine was stopped on account of acute infection, as three-lineage cytopenia was detected on admission: her white blood cell count was 2.5 (4.5–11 × 10^9^/L), platelets 38 (200–450 × 10^9^/L) and hemoglobin 89 g/L (117–155 g/L). Splenomegaly, acute cardiac failure and acute hepatic failure, with 15% necrosis in a biopsy, were observed in the next few days. Symptoms of encephalitis were present, but a brain MRI was normal. Viral genome examinations by EBV polymerase chain reaction revealed > 350,000 copies/mL in plasma on admission and > 1 million copies/mL after 4 days of treatment (Fig. 1). A high viral load of > 8 million copies/mL was also detected in the liver biopsy specimen. Tests for IgM antibodies to VCA were positive on admission, whereas IgG antibodies to EBNA were negative, indicating acute EBV infection. She was treated once with rituximab and then with corticosteroids, acyclovir and intravenous immunoglobulin. She developed severe multiple organ failure, including cardiac, hepatic and respiratory failure, and was critically ill for 4 weeks in the intensive care unit. After this treatment in the intensive care unit, approximately 30 days after admission, the viral load started to increase again and she developed signs of severe hepatitis and pneumonitis, so that regular weekly rituximab infusions were given for a total of 8 weeks. Very low NK-cells were noted and the persistent viremia (detection level 300 copies/mL) stopped when the NK-cell count started to rise, approximately 90 days after the cessation of azathioprine (Fig. 1). During the course of illness, CD8+ T-cell counts were 1131 (220–1129 × 10^6^/L) on day 32, 2535 on day 70, 2487 on day 93 and 2537 on day 106. Due to the prolongation of viremia and hepatitis despite the discontinuation of azathioprine, WES was performed. This did not reveal any known primary immunodeficiency, but the patient was found to be carrying two rare heterozygous variants of the *TLR3* and *OAS1* genes, in which variants have previously shown to be associated with susceptibility to viral infections. TPMT genotype leading to low enzyme activity was excluded in this case after the patient’s recovery. A high total IgG level (> 15 g/L) persisted for 6 months after clearance of the viremia, and the low CD19-cell count attributable to the rituximab treatment started to rise 6 months after cessation of the infusions. At the last follow-up visit, she was asymptomatic and healthy and had successfully returned to her studies 6 months after the onset of the EBV infection. She was still in remission with respect to her inflammatory bowel disease without any medication.

### Systematic review of the literature

Examination of the 13 relevant publications yielded a total of 17 corresponding cases, mainly of adolescents or young adults with Crohn’s disease or ulcerative colitis who had been treated with azathioprine and developed either severe or fatal EBV infections, or EBV-driven hemophagocytic lymphohistiocytosis or some other lymphoproliferative disorder, excluding lymphomas [3–15]. Four patients had died [4, 7, 10, 14]. High EBV viremia (> 100,000 copies/mL) was documented in five patients [3, 7, 10–12]. NK-cell counts had been made in two out of the 17 patients and either a low count or low NK-cell cytotoxicity had been noted during the acute phase of the illness in both patient cases [3, 13]. CD8+ T-cell counts of the patients were not reported in the literature. Three patients had been successfully treated with repeated rituximab infusions [3, 8, 11]. Most patients had received corticosteroids and also acyclovir or ganciclovir. X-linked lymphoproliferative disease (XLP) was excluded in two male patients by genetic testing [7, 10].

## Discussion and conclusions

Azathioprine is widely used for the treatment of Crohn’s disease and ulcerative colitis. Our case and systematic literature search confirm that a primary EBV infection can be extremely severe or even fatal for EBV-negative adolescents or young adults receiving azathioprine.

Azathioprine has recently been shown to cause rather specific immunosuppression, resulting in NK-cell [16, 17], but not T-cell depletion [17]. Moreover, early-differentiated NK-cells seem to be critical in the immune control of primary EBV infection as this subset of NK-cells expands and restricts lytic EBV infection that is poorly controlled in infectious mononucleosis [18, 19]. We repeatedly monitored our patient’s lymphocyte subpopulation count using flow cytometry and noted an almost total loss of the NK-cell population, whereas the number of CD8+ T-cells were within the normal range. We were thus able to draw a clear time series showing that the clearance of EBV viremia coincided with the normalization of NK-cell counts three to four months after ceasing to administer azathioprine. This supports the idea that azathioprine caused a severe secondary immunodeficiency and a lack of NK-cells, resulting in an uncontrolled EBV infection and hyperinflammation, since NK-cells play a role in down-regulating inflammatory responses [20]. Thus the phenotype observed in our patient mimics the known pathomechanism of congenital hemophagocytic lymphohistiocytosis, which is a hyperinflammatory condition explained by low cytotoxic functioning of the NK-cells or T-cells on account of genetic defects [20]. The risk of hemophagocytic lymphohistiocytosis in children with Crohn’s disease, often treated with thiopurines, has been estimated to be 100-fold in a population-based patient series of 5 cases with HLH and inflammatory bowel disease [4]. The risk of lymphoproliferative disorders, including lymphomas, has been reported in a large cohort of inflammatory bowel disease patients to be five-fold in those patients receiving thiopurines [21]. Our findings offer a possible explanation for this observed epidemiological risk.

Corticosteroids and acyclovir are recommended for the treatment of severe EBV infection. After an organ or HSCT EBV is known to cause a lymphoproliferative disorder which can be treated with rituximab, an anti-CD20 monoclonal antibody, in order to reduce B-cells and stop viremia, since B-cells are the main replication site of Epstein-Barr viruses during infection. In patients with XLP, a primary immunodeficiency leading to uncontrolled EBV infection, severe EBV infection has recently been treated with rituximab before HSCT. We chose to treat our patient with corticosteroids, acyclovir and one dose of rituximab at the onset of the disease. However, as the EBV viral load started to increase again after 1 month despite corticosteroid and acyclovir therapy and she simultaneously developed new signs of severe pneumonitis and hepatitis, we decided to administer rituximab weekly until viremia had been arrested, three months after admission. Donor-derived EBV-specific cytotoxic T-cells are a novel experimental treatment for uncontrolled EBV infections in patients with primary or secondary immunodeficiency [22], and we consequently considered treating our patient with EBV-specific cytotoxic T-cells before she started to respond well to rituximab treatment.

The previous cases reported in the literature had not undergone thorough WES to rule out undiagnosed known primary immunodeficiencies, and even though XLP was not a diagnostic alternative for a female patient, defects in the *ITK*, *STK4*, *TNFRSF7* and *CORO1A* genes, for instance, can lead to a life-threatening EBV infection. If a primary immunodeficiency had been diagnosed, the patient could have been considered for treatment with EBV-specific cytotoxic T-cells as a bridge before HSCT. Rapid WES is thus a very useful diagnostic tool in cases of unusually severe EBV infection, in order to exclude primary immunodeficiencies, as these need to be treated with a prompt HSCT.

Our clinical case demonstrates that azathioprine-induced NK-cell depletion could be the reason for a severe, life-threatening primary EBV infection in this context. Achieving immune reconstitution may take several months after the cessation of azathioprine medication. All infectious disease specialists treating adolescents and young adult patients who have been receiving azathioprine should be aware of the risk. Testing for EBV serology before starting azathioprine could help to identify EBV-seronegative adolescents and young adults who might have a high risk of developing an unusually severe primary infection.

## Abbreviations

EBV: Epstein-Barr virus
EBNA: Epstein-Barr virus nuclear antigen
HSCT: Hematopoietic stem cell transplantation
Ig: Immunoglobulin
NK: Natural killer
VCA: Viral capsid antigen
WES: Whole exome sequencing
